# Microbial Oligosaccharides with Biomedical Applications

**DOI:** 10.3390/md19060350

**Published:** 2021-06-21

**Authors:** Jian-Lin Xu, Zhi-Feng Liu, Xiao-Wei Zhang, Hai-Li Liu, Yong Wang

**Affiliations:** 1 Key Laboratory of Synthetic Biology, CAS Center for Excellence in Molecular Plant Sciences, Institute of Plant Physiology and Ecology, Chinese Academy of Sciences, Shanghai 200032, China; xujianlin@cemps.ac.cn (J.-L.X.); liuzhifeng@cemps.ac.cn (Z.-F.L.); xiaoweizhang@cemps.ac.cn (X.-W.Z.); 2 University of Chinese Academy of Sciences, Beijing 100039, China; 3 State Key Laboratory of Bioreactor Engineering, East China University of Science and Technology, Shanghai 200237, China

**Keywords:** microbial oligosaccharide, aminooligosaccharide, saccharomicin, orthosomycin, chemical structure, biomedical applications, biosynthesis

## Abstract

Microbial oligosaccharides have been regarded as one of the most appealing natural products attributable to their potent and selective bioactivities, such as antimicrobial activity, inhibition of α-glucosidases and lipase, interference of cellular recognition and signal transduction, and disruption of cell wall biosynthesis. Accordingly, a handful of bioactive oligosaccharides have been developed for the treatment of bacterial infections and type II diabetes mellitus. Given that naturally occurring oligosaccharides have increasingly gained recognition in recent years, a comprehensive review is needed. The current review highlights the chemical structures, biological activities and divergent biosynthetic origins of three subgroups of oligomers including the acarviosine-containing oligosaccharides, saccharomicins, and orthosomycins.

## 1. Introduction

Oligosaccharide chains are ubiquitous across the natural world, which can be found in plants, marine algae, and microorganisms, especially the filamentous bacteria of the order of Actinomycetes. Despite the majority of oligosaccharide chains are present in glycoproteins and glycolipids, they are also found in natural products. It has been demonstrated that the formation of oligosaccharide chains is a fundamental determinant of bioactivity for many secondary metabolites, which is capable of improving or broadening their therapeutic potential [1,2,3,4].

Natural oligosaccharides are a class of carbohydrates polymers, which are normally composed of two to ten monosaccharide units linked together through glycosidic bonds [5]. Due to the diversity of the monomeric sugars and modified side chains and their flexible sequences of assembly, oligosaccharide natural products tend to possess an abundant structural skeleton, which can be divided into four categories based on their core structures including aminooligosaccharides, saccharomicins, orthosomycins, and moenomycins. Most oligosaccharides have been proved to exert a crucial influence in various biological processes, such as signal transduction, molecular recognition, inhibition of α-glucosidases and lipase, and inhibition of pathogenic bacterial growth. Therefore, a handful of bioactive oligosaccharides have been widely used for the treatment of diseases in humans, animals, and plants [6,7,8].

Nevertheless, the oligomeric natural products are less prevalent than other classes of compounds and their biosynthesis has not received sufficient attention in the last century, presumably attributable to their relatively complex and heterogeneous structures, trace-level compositions, and less stable backbones, which is more difficult to detect and identify. In the past two decades, the development of liquid chromatography/high-resolution mass spectrometry (LC/HRMS) has brought considerable progress for the structural analysis of oligosaccharides, which enables users to obtain the detailed primary and secondary fragment ions of oligosaccharides including molecular formula, sugar constituent, and the position of the side chains [9,10,11,12]. An increasing number of oligosaccharides with significant biological properties have been rapidly identified with the aid of LC-HRMS technology, which has attracted much attention in studying their unique biosynthetic pathway and developing potential lead compounds. However, the only review with respect to oligosaccharides was published by Emilianne K. McCranie and Brian O. Bachmann in 2014, which described the mode of action and biosynthesis of bioactive oligosaccharide natural products [13]. In recent years, quite a few new members of bioactive oligosaccharide have been reported, but there is no additional review that updates the structures and biosynthesis of this family of natural products.

In the current review, we focus on the chemical structures and biological activities of oligosaccharide natural products isolated from a microorganic system. A total of 99 compounds including 39 aminooligosaccharides, 2 saccharomicins, and 58 orthosomycins have been summarized. Meanwhile, the recent progress of the biosynthetic mechanism of the representative oligomeric metabolites is highlighted, which contributes to discover new oligosaccharides with the aid of genomic mining and enables to provide opportunities to generate oligosaccharides with improved pharmacological properties through rational reengineering. In addition, the mechanisms of action of three oligosaccharides (aminooligosaccharides, saccharomicins, and orthosomycins) were described in detail by the Emilianne K. McCranie and Brian O. Bachmann. Thus, this review will briefly summarize the mode of action of the representative oligosaccharides with new progresses.

## 2. Acarviosine-Containing Aminooligosaccharides

### 2.1. Structural Features and Biological Properties

The acarviosine refers to the pseudo-disaccharide core that is composed of an unsaturated C_7_N cyclohexitol residue (valienamine) and a 4-amino-4,6-dideoxy-*d*-glucopyranose (4-amino-4-deoxy-*d*-quinovopyranose) unit. The acarviosine-containing aminooligosaccharides is a relatively new class of natural products exclusively produced by microorganisms, which usually possess one or more pseudo-trisaccharide moiety (moieties) formed by an acarviosine unit and a *d*-glucopyranose group through an α-(1 → 4) quinovosidic bond, which are further combined with *d*-glucose units attached to the reducing and non-reducing end through an α-(1 → 4) glycosidic bond [14].

**Figure 1:**
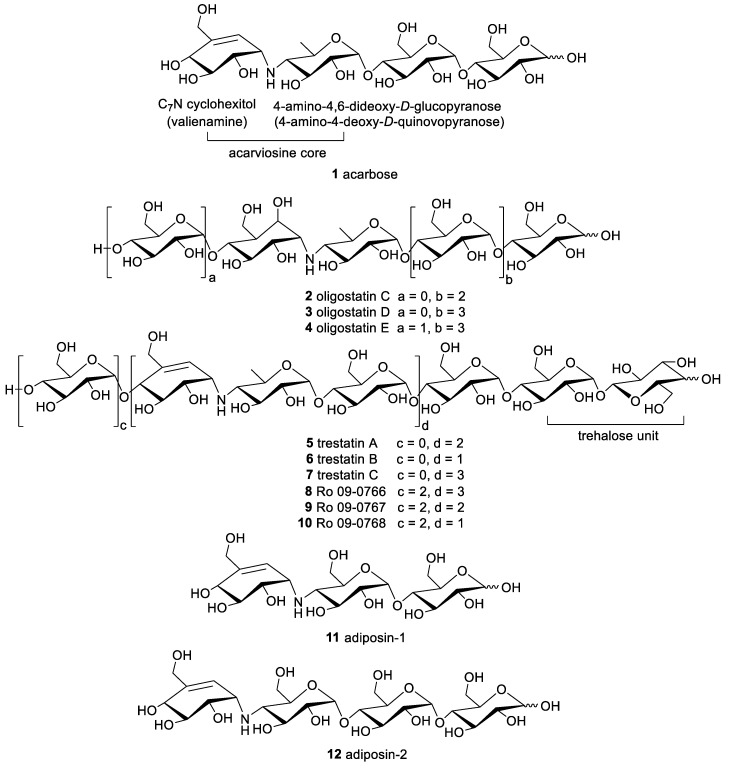
The chemical structures of compounds **1**–**12**.

In the course of the screening for new α-glucosidase inhibitors derived from microorganism, the pseudo-oligosaccharidic complex with significant inhibitory effect against α-glucosidases, especially α-amylases and sucrase, was discovered in the culture filtrates of the *Actinoplanes* strain SE 50. The pseudotetrasaccharide acarbose (**1**), the major and most active component of the complex, was reported by Schmidt and his coworkers in 1977 (Figure 1), which exhibited conspicuous inhibitory activity against glucoamylase, sucrase, maltase, and dextrinase [15,16]. This represents the first reported example of acarviosine-containing compound. Structurally, acarbose is composed of an unsaturated C_7_N cyclohexitol, a deoxyhexose, and a maltose. The pivotal substructure acarviosine, consisting of the valienamine and the deoxyhexose, has been postulated to be indispensable for its enzyme inhibitory activity. Since then, the continued research and new discoveries of homologous natural products acquired from the microorganism have been published.

Large-scale fermentation of *Streptomyces myxogenes* nov. sp. SF-1130 yielded three new metabolites oligostatins C–E (**2**–**4**), which represent the saturated pseudo-penta, pseudo-hexa, and pseudo-heptasaccharides fused with a nitrogen containing glycosidic bond. In contrast to the α-glucosidase inhibitors previously reported, the olefinic bond in valienamine moiety was reduced to hydroxy forms, which was determined by spectroscopic analysis and chemical degradation. In addition to the pronounced α-amylase inhibitory activity, these compounds also showed antimicrobial activity towards several Gram-negative bacteria [17,18]. The chemical investigation of *Streptomyces dimorphogenes* NR320-OM7HB led to the isolation of six components named trestatins A–C (**5**–**7**), Ro 09-0766 (**8**), Ro 09-0767 (**9**), and Ro 09-0768 (**10**), all of which have a unique trehalose residue. These compounds exhibited pronounced α-amylase inhibitory activity with the IC_50_ values ranging from 8.1 to 610 nM [19,20,21]. Additional research on another bacterial strain *Streptomyces calvus* TM-521 collected in Tokyo produced two oligosaccharides adiposin-1 (**11**) and adiposin-2 (**12**). Adiposin-2 was nearly identical with acarbose, except for the replacement of deoxyglucose moiety in acarbose by the glucose ring, which was further supported by the acid hydrolysate of the adiposin complex [22,23,24,25].

**Figure 2:**
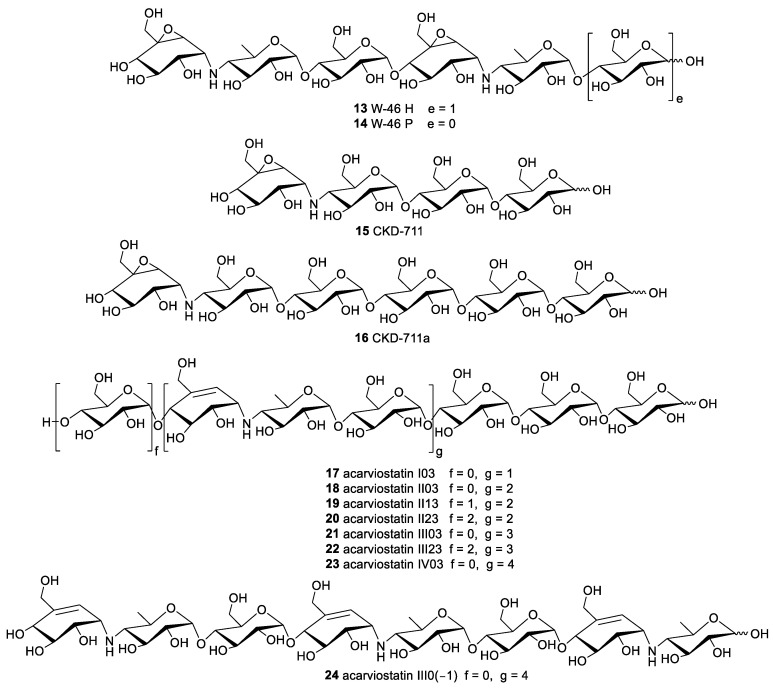
The chemical structures of compounds **13**–**24**.

Two new oxirane pseudooligosaccharides carrying two epoxide-containing C_7_N aminocyclitols were identified from *Streptomyces galbus* subsp. FH 1716 (DSM 3007) and given the trivial name W-46 H and W-46 P (**13** and **14**) [8,26]. Another two congeners CKD-711 and CKD-711a (**15** and **16**), sharing one epoxide-pseudoaminosugar unit, were acquired from the soil actinomycete *Streptomyces* sp. CK-4416 (Figure 2). Compound **15** showed stronger inhibitory activity than its hexameric analog **16** against sucrase, maltase, and α-amylase with IC_50_ values of 0.5, 2.5, and 78.0 μg/mL, respectively, indicating that multiple glucose units might pose a detrimental effect for bioactivities. Simultaneously, the moderate of antibacterial activity against *Comamonas terrigena* was reported for compounds **15** and **16** [27,28,29].

The relatively abundant subgroup of natural aminooligosaccharides is the acarviostatins, which is generally termed as acarviostatins followed by a Roman numeral and two numbers. The Roman numeral denotes the number of the pseudo-trisaccharide cores; the middle digit represents the number of glucose residues at the non-reducing end; and the last digit corresponds to the number of glucose units at the reducing end. The arrangement and assembly of the repeating pseudotrisaccharide units with different numbers of *d*-glucopyranose at the reducing and non-reducing termini yields numerous acarviostatins. A total of eight acarviostatins (**17**–**24**) were initially identified from the mixed extract of *Streptomyces coelicoflavus* ZG0656, all of which were mixed noncompetitive inhibitors of porcine pancreatic α-amylase (PPA) with the inhibition constants (Ki) ranging from 0.008 to 0.346 μM [30,31,32].

**Figure 3:**
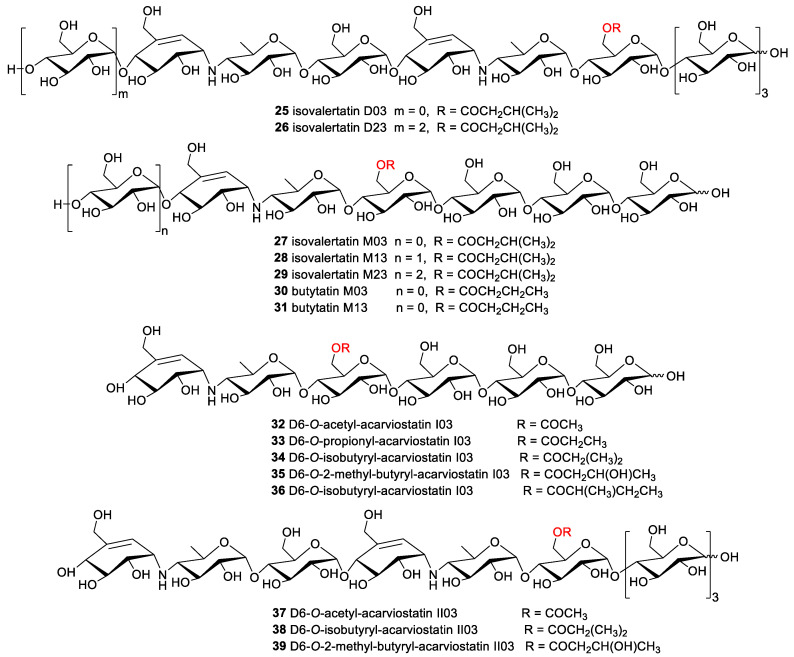
The chemical structures of compounds **25**–**39**.

The discovery of the acylated acarviostatins has expanded the structural diversity of this family of oligosaccharide. To the best of our knowledge, fifteen acylated aminooligosaccharides have been separated from two different *Streptomyces* strains. Although five isovalerylated aminooligosaccharides had been purified from culture broths of the soil *Streptomyces luteogriseus* in 1995, there were several inconsistencies against their corresponding electrospray ionization mass spectrometry (ESIMS) when the researchers rechecked their structures. The extensive spectroscopic analyses including ESI multistage mass spectrometry, 2D NMR technique and chemical conversion resulted in their structural revision, and these oligomers were redesignated as isovalertatins D03 (**25**), D23 (**26**), M03 (**27**), M13 (**28**), and M23 (**29**) (Figure 3). This is the first report of the acylated acarviostatins sharing an isovaleryl group at C-D6 [33,34]. At nearly the same time that the structures of isovalertatins were revised, two butylated congeners butytatins M03 and M13 (**30** and **31**) were isolated from the same *Streptomyces* strain [35]. Additional investigation on marine-derived *Streptomyces* sp. HO1518, collected in Yellow Sea, yielded eight new acylated acarviostatins (**32**–**39**) and two known analogues (**18** and **25**). All of them showed promising inhibitory activities against both α-glucosidase and lipase enzymes under the low micromolar concentrations [36,37].

As mentioned above, all of acarviosine-containing aminooligosaccharides exhibited potent inhibitory activities against key digestive enzymes involved in the breakdown of polysaccharides, such as α-amylase, sucrase, maltase, and isomaltase. Much of the research revealed that the number of the pseudo-trisaccharide core and glucose unit attached to the reducing and non-reducing end played a pivotal role in the inhibition potency of digestive enzymes [32,38]. Generally speaking, aminooligosaccharides containing two or three pseudo-trisaccharide units tend to exhibit the most potent α-glucosidase inhibitory activities, among which the inhibitory potencies of aminooligosaccharides with nine to twelve sugar rings is maximally effective. For instance, *D*6-*O*-acetyl-acarviostatin II03 (**37**) possessing two pseudo-trisaccharides was the most potent α-amylase and sucrase inhibitor, with the IC_50_ values 540-fold and 27-fold stronger than acarbose. In addition, eight acylated aminooligosaccharides (**32**–**39**) were detected to possess significant inhibitory effect against pancreatic lipase, amongst which *D*6-*O*-isobutyryl-acarviostatin II03 (**38**) sharing two pseudo-trisaccharide units was the most active lipase inhibitor with the IC_50_ value of 0.82 μM. It is worth noting that the acylated aminooligosaccharides with lipase inhibitory activity were reported for the first time. Although acarbose failed to show any antibacterial activity, some of aminooligosaccharides had notable inhibitory effects on the growth of various pathogenic bacteria. For example, oligostatins C–E (**2**–**4**) were active against Gram negative bacteria, e.g., *Shigella sonnei*, *Escherichia coli*, *Salmonella typhi*, *Proteus vulgaris*, and *Klebsiella pneumonia*. Adiposin-2 (**12**), which closely resembled acarbose, except for the replacement of deoxyglucose in acarbose by the glucose moiety adjacent to the core unit valienamine, exhibited antibacterial activity with some Gram positive bacteria, Gram negative bacteria, and phytopathogenic fungi. Interestingly, when the double bond of valienamine is tailored into its epoxy form in aminooligosaccharides, their antibacterial activity will be altered. The epoxidal aminooligosaccharides CKD-711 and CKD-711a (**15** and **16**) displayed a highly selective antibacterial activity against *Comamonas terrigena* ATCC 8461 instead of the Gram negative and Gram positive bacteria tested by comparison with the oligostatins and the adiposins.

### 2.2. Mode of Action

In 2011, the structures of human pancreatic α-amylase (HPA) in complex with acarviostatins clearly revealed the mode of action of acarviostatins. Consistent with acarbose, acarviostatin I03 underwent hydrolysis, condensation, and transglycosylation reactions, which led to a six-ring rearranged product acarviostatin I21 that bound tightly in the active site of HPA. Quite interestingly, the larger acarviostatins containing repeated pseudo-trisaccharide cores (acarviostatins II03, III03, and IV03) might undergo only hydrolysis reactions, resulting in the generation of the same seven-ring modified product in the HPA active site. The authors demonstrated that the hydrolysis product with seven sugar rings would show the most efficient α-amylase inhibition attributable to the occupation of the full active site [39,40].

### 2.3. The Biosynthesis of Acarbose and the Acarviostatins

**Figure 4:**
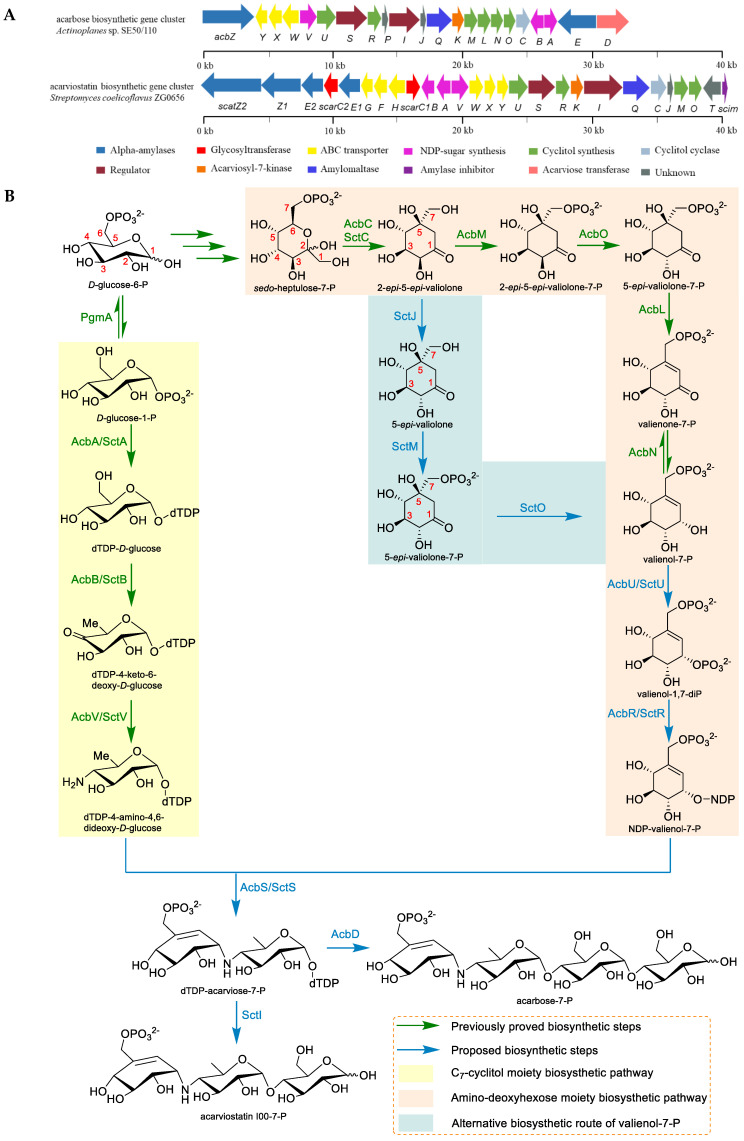

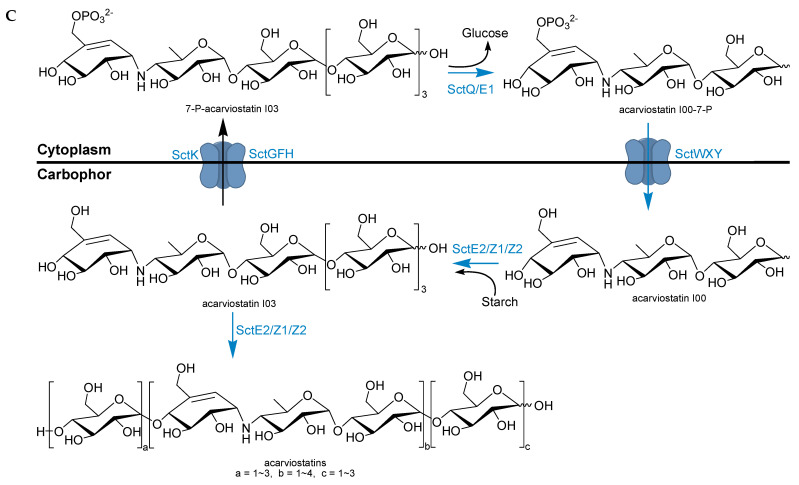
The gene cluster and biosynthesis of acarbose and acarviostatins. (**A**) Comparison between acarbose biosynthetic gene cluster (*acb*-cluster) of *Actinoplanes* sp. SE50/110 and acarviostatin biosynthetic gene cluster (*sct*-cluster) of *Streptomyces coelicoflavus* ZG0656. The functions of eight genes in the *acb*-cluster (AcbA, AcbB, AcbV, AcbC, AcbM, AcbO, AcbL, and AcbN) are experimentally defined by proteomic studies, while the functions of the remaining genes in *acb*-cluster and all genes in the sct-cluster are proposed by bioinformatic analyses. (**B**) The proposed intracellular biosynthetic pathway of acarbose in *Actinoplanes* sp. SE50/110 and acarviostatin in *Streptomyces coelicoflavus* ZG0656. (**C**) The proposed extracellular biosynthesis of acarviostatins and the carbophor in *Streptomyces coelicoflavus* ZG0656.

The acarbose and acarviostatins are considered to be the representative members of the aminooligosaccharides family, and their biosynthesis will be briefly summarized in this review. Two known biosynthetic gene clusters of acarbose termed *acb*-cluster and *gac*-cluster were identified from *Actinoplanes* sp. SE50/110 and *Streptomyces glaucescens* GLA.O, respectively; whereas the presumptive acarviostatin gene cluster (*sct*-cluster) was revealed in the *Streptomyces coelicoflavus* ZG0656 in 2012 (Figure 4). The *sct*-cluster shows high similarity to the *acb*-cluster, especially several similar structural genes (SctA, SctB, SctC, SctS, SctR, SctU, and SctV) encoding the synthesis of pseudodisaccharidic acarviosyl moiety and ATP-dependent transporter system (SctW, SctX, and SctY) (Table 1). Consequently, in analogy to the acarbose biosynthetic pathway, the biosynthesis of the acarviostatins has been postulated based on the current knowledge of acarbose biosynthesis and the functional annotation of each synthetic gene [41,42,43].

Biochemically, the biosynthesis of valienol-7-phosphate (valienol-7-P) of the acarviostatins is somewhat different from that of acarbose. The origin of the valienamine moiety in acarbose has been established by Floss and coworkers via labeling experiments, which is derived from the pentose phosphate pathway [44]. Then, Piepersberg and his colleagues have demonstrated that the precursor *sedo*-heptulose-7-phosphate (*sedo*-heptulose-7-P) is cyclized by the cyclase (AcbC/SctC) to produce 2-*epi*-5-*epi*-valiolone, which is then phosphorylated by the phosphotransferase (AcbM) and catalyzed by the isomerase (AcbO) to generate intermediate 5-*epi*-valiolone-7-P [45,46,47]. The cyclitol dehydrogenase (AcbL) has been proven to catalyze the dehydration at C-5 and C-6 of 5-*epi*-valiolone-7-P to yield valienone-7-P, which is further modified by the NADPH-dependent oxidoreductase (AcbN) to give valienol-7-P [48]. Due to the lack of homologs of the genes AcbO, AcbL, and AcbN within the *sct* biosynthetic gene clusters, two unique genes SctJ and SctO in the *Streptomyces coelicoflavus* ZG0656, together with the gene SctM similar to AcbM, are putatively responsible for an alternative biosynthesis route of valienol-7-P. The intermediate 2-*epi*-5-*epi*-valiolone in the pathway of the acarviostatins is proposed to be epimerized by SctJ, which is phosphorylated and dehydrated through the sequential action of SctM and SctO, leading to generate valienol-7-P [43]. The remaining biosynthesis of the pseudodisaccharidic acarviosyl moiety of the acarviostatins is analogous to those of acarbose. It is speculated that AcbU/SctU should be responsible for the second phosphorylation of valienol-7-P to obtain valienol-1,7-diP, and the latter is converted to NDP-valienol-7-P with the aid of AcbR/SctR.

On the other hand, the biosynthesis of the amino-deoxyhexose moiety is derived from *d*-glucose-1-P as the initial precursor, which is successively catalyzed by AcbA/SctA (dTDP-glucose synthase), AcbB/SctB (4,6-dehydratase), and AcbV/SctV (4-aminotransferase), leading to produce dTDP-4-amino-4,6-dideoxy-*d*-glucose [49,50]. The resulting amino-deoxyhexose is proposed to converge with C_7_-cyclitol by AcbS/SctS (glycosyltransferase), which synthesizes pseudodisaccharidic acarviosyl moiety dTDP-acarviose-7-P. The dTDP-acarviose-7-P has been proposed to involve in the glycosylation coded by AcbD, an exclusive acarviose transferase in the *acb*-cluster that adds a maltose to the substrate to form acarbose-7-P [51]. The transporter system AcbWXY, which is predicted to participate in a recycling mechanism, secretes acarbose-7-P out of the cell and then is dephosphorylated to obtain acarbose. In the biosynthetic pathway for acarviostatins, the glycosyltransferase SctI is predicted to mediate the transformation of phosphorylated pseudodisaccharide into phosphorylated pseudotrisaccharide acarviostatin I00-7-P. Similarly, the putative transporter SctWXY homologous to AcbWXY is accountable for the export of acarviostatin I00-7-P, resulting in the active pseudotrisaccharide core acarviostatin I00. Three extracellular α-amylases (SctE2, Z1, and Z2) may digest starch into different lengths of maltodextrins and connect these maltooligosaccharides to acarviostatin I00. The resulting acarviostatin homologues are reimported back into the cytoplasm by the transporter SctFGH, which is phosphorylated by the kinase SctK. Then, SctQ and SctE1 hydrolyze the glucoses residues attached to pseudotrisaccharide to regenerate acarviostatin I00-7-P. Thereby, the researchers speculate that the strain ZG0656 is capable of transporting hydrolysates of starch into the cell with the use of this recycling mechanism, which may also explain a plethora of acarviostatins, which have been detected in the host [43,52].

Taken together, a putative biosynthetic pathway of acarviostatins including intracellular assembly and extracellular extension has been proposed on the basis of bioinformatic analysis. However, the biochemical functions of every enzyme in the *sct*-cluster have not been experimentally characterized thus far, which makes it difficult to identify critical biosynthetic bottlenecks of the acarviostatins and increase their titer through metabolic engineering. The acarviostatins, especially the acylated derivatives, are recognized as a subgroup of promising oligosaccharide natural products attributable to their remarkable inhibitory activities against both α-glucosidase and lipase, which has aroused our medical interest to study the biosynthesis of the acylated acarviostatins. The biosynthesis of backbone structure and the assembly of acyl side chains will be revealed in the further studies. Fully understanding the biosynthesis of the acylated acarviostatins contributes to discover new analogues with improved curative effect that have the potential to fight against diabetes and obesity.

## 3. Saccharomicins

### 3.1. Structural Features and Biological Properties

**Figure 5:**
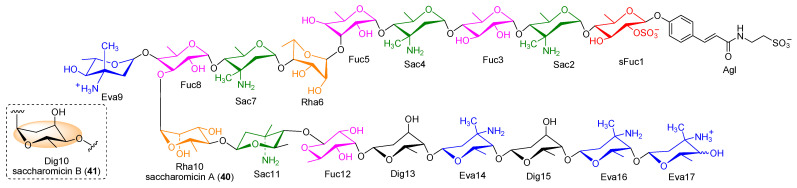
The chemical structures of saccharomicins A and B (**40** and **41**). Agl: aglycone; sFuc: sulfated fucose; Sac: saccharosamine; Fuc: fucose; Rha: rhamnose; Eva: 4-*epi*-vancosamine; Dig: digitoxose.

A novel class of structurally intriguing oligosaccharides were isolated in 1998 from the mixed extract of a rare actinomycete *Saccharothrix espanaensis* LL-C19004 and termed as the saccharomicins. Only two saccharomicins named saccharomicins A and B (**40** and **41**) have been reported until now (Figure 5), and their subtle difference of the structure was found in the tenth sugar residue, where the α-rhamnopyranosyl in **40** was replaced by the α-digitoxopyranosyl in **41**. Saccharomicins are constructed by a *N*-(*m*,*p*-dihydroxycinnamoyl) taurine aglycone and seventeen 6-deoxy sugars, including a sulfated fucose, four fucoses, four saccharosamines, four 4-*epi*-vancosamines, and four of a combination of rhamnose(s) and digitoxoses. Interestingly, the fucoses are always coupled with saccharosamines through a unique *β*-*β* glycosidic bond, while the glycosidic linkages of digitoxoses are determined as α-configuration. In contrast with all known families of antibiotics, saccharomicins belong to new heptadecaglycoside antibiotics, which displayed conspicuous antibacterial activity against the Gram-positive pathogenic bacteria tested, in particular for all multiply drug-resistant staphylococci strains. In addition, both saccharomicins A and B also exhibited activity, despite it being to a relatively lower extent, against Gram-negative microorganisms [53,54]. These results implied that this unprecedented class of oligosaccharides might have the potential to be utilized as an antibacterial drug candidate, which has immensely fascinated pharmaceutical scientists to conduct extensive research efforts toward their biosynthesis.

### 3.2. Mode of Action

Until now, the precise mechanism of action of the saccharomicins has not been determined. The mechanistic investigations with *Escherichia coli* and *Bacillus subtilis* implied that the primary cellular target of the saccharomicins is associated with the bacterial membrane. Within 10 min of saccharomicin A treatment, the complete, nonspecific inhibitory effect of DNA, RNA, and protein biosynthesis was observed, which indicated that the cell membrane integrity in the bacteria was disrupted. Moreover, the extensive leakage of intracellular potassium from *E. coli* also revealed the membrane-damaging effects of saccharomicin A, which would lead to bacterial cell lysis [54].

### 3.3. The Biosynthesis of Saccharomicin A

**Figure 6:**
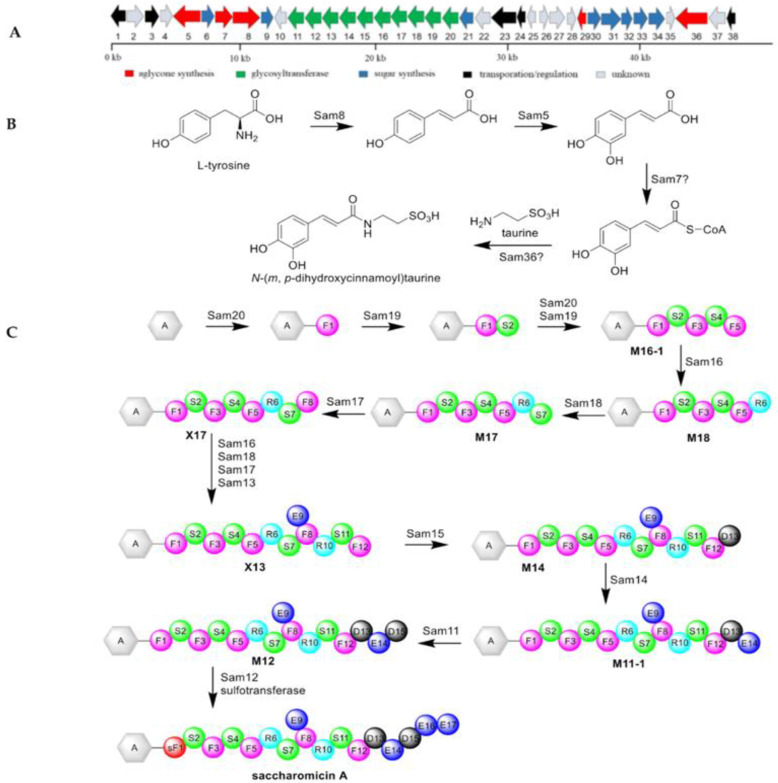
The gene cluster and biosynthesis of saccharomicin A. (**A**) The biosynthetic gene cluster of saccharomicin A. The functions of twelve genes in the saccharomicin A biosynthetic gene cluster (Sam5, Sam8, and Sam11-20) are experimentally defined by proteomic studies, while the functions of the remaining genes are proposed by bioinformatic analyses. (**B**) Biosynthetic pathway of the saccharomicin aglycone. (**C**) The proposed glycosylation pathway in saccharomicin A biosynthesis.

In 2012, the genome sequence of *S. espanaensis* DSM 44229^T^ revealed the presence of the saccharomicin biosynthetic gene cluster, which was designated as the *sam* cluster. This gene cluster harbors five genes necessary for the formation of the aglycone substructure, and eight genes responsible for the production of NDP-sugar precursors. There are ten putative glycosyltransferases (Sam11-20) in the cluster, which is in charge of the assembly of the heptadecasaccharide chain, indicating that several of glycosyltransferases should work iteratively (Figure 6) [55].

Bechthold and colleagues first reported the biosynthesis of the aglycone moiety of the saccharomicins in 2006. They identified the biochemical function of two candidate genes (Sam5 and Sam8) derived from *S. espanaensis*, which were accountable for caffeic acid biosynthesis. Heterologous expression of Sam8 led to the formation of *trans*-*p*-coumaric acid, while coexpression of Sam8 and Sam5 resulted in the production of *trans*-caffeic acid. The enzyme assay unambiguously revealed that the natural substrate of Sam8 was l-tyrosine rather than l-phenylalanine, simultaneously demonstrating that Sam8 was a tyrosine ammonia-lyase and Sam5 was a 4-coumarate 3-hydroxylase [56]. The gene Sam7 shows similarity to acyl-CoA synthetases, which is thought to catalyze the linkage between caffeate and CoA, resulting in the corresponding thioester caffeoyl-CoA. The product may be fused with taurine to form the entire aglycon via Sam36 that has homology to penicillin amidases [55].

More recently, the assembling mechanism of the heptadecasaccharide chain of saccharomicin A has been reported. Assembly of the saccharide chain has been demonstrated to begin from the reaction of Sam20, which transfers a Fuc to the *p*-phenol group of aglycone. Sam19 transfers a Sac to the C4-hydroxyl of the first nascent Fuc, which is catalyzed by the cooperative action of Sam20 and Sam19 to form pentaglycan **M16-1**. Subsequently, a Rha is attached to the pentasaccharide chain **M16-1** by Sam16 to obtain **M18**, which is further modified by the Sac transferase (Sam18) and Fuc transferase (Sam17) to give octaglycoside **X17**. The saccharide chain is elongated by the repeated glycosylation encoded by Sam16, Sam18, Sam17, and Sam13, resulting in the formation of dodecaglycoside **X13**. The latter is sequentially glycosylated by the Sam15-catalyzed Dig transfer and the Sam14-catalyzed Eva transfer, which is modified by the Dig transferase (Sam11) to form M12. The last two Eva residues are incorporated into M12 under the repeated actions of Sam12. Finally, authors hypothesize that sulfation of Fuc1 is predominantly completed after the assembly of the oligosaccharide chain, which is attributed to the existence of the sulfated Fuc (sFuc) in heptadecaglycoside [57].

This study uncovered the glycosylation process in the biosynthetic network of the heptadecaglycoside saccharomicin A and described functional dissection of every glycosyltransferase in detail, which would be beneficial to fully understand the biosynthesis of the oligosaccharide antibiotic containing the longest saccharide chain existed in natural products. Meanwhile, it also expanded the ever-increasing knowledge on the biosynthesis of oligomeric secondary metabolites and set the stage for pathway engineering of the saccharomicins and discovery of new drug candidates.

## 4. Orthosomycins

### 4.1. Structural Features and Biological Properties

Orthosomycins are recognized as a new family of oligosaccharide antibiotics, which are hallmarked by the presence of one or more unique interglycosidic spirocyclic ortho-*δ*-lactone (orthoester) linkage(s) between sugar residues. It is generally acknowledged that the rarely observed orthoester linkage is necessary for the antibiotic properties of the orthosomycins. Although the orthosomycin family includes hygromycin B, everninomicin, avilamycin, flambamycin, curamycin, etc., the former three members have been extensively studied, especially in the aspect of their bioactivity and biosynthesis [58,59,60,61,62,63,64,65,66,67,68,69,70,71,72,73,74,75,76,77,78,79,80,81,82]. Herein, restricted by the space, everninomicin (EVN) and avilamycin (AVI) will be mainly discussed in this review.

**Figure 7:**
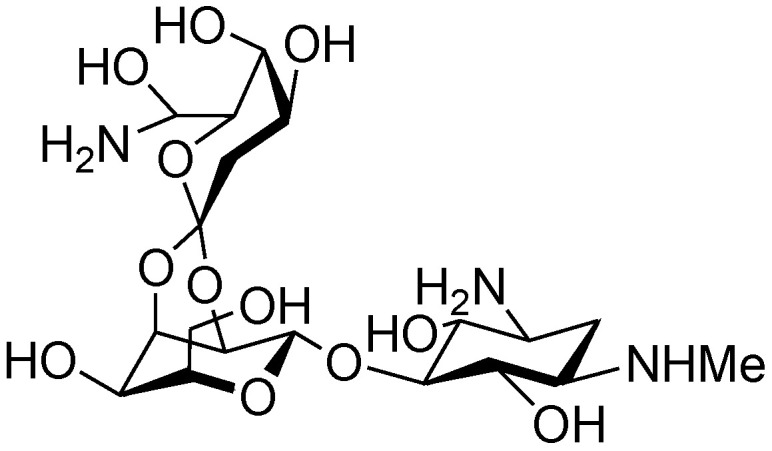
The chemical structure of hygromycin B (**42**).

**Figure 8:**
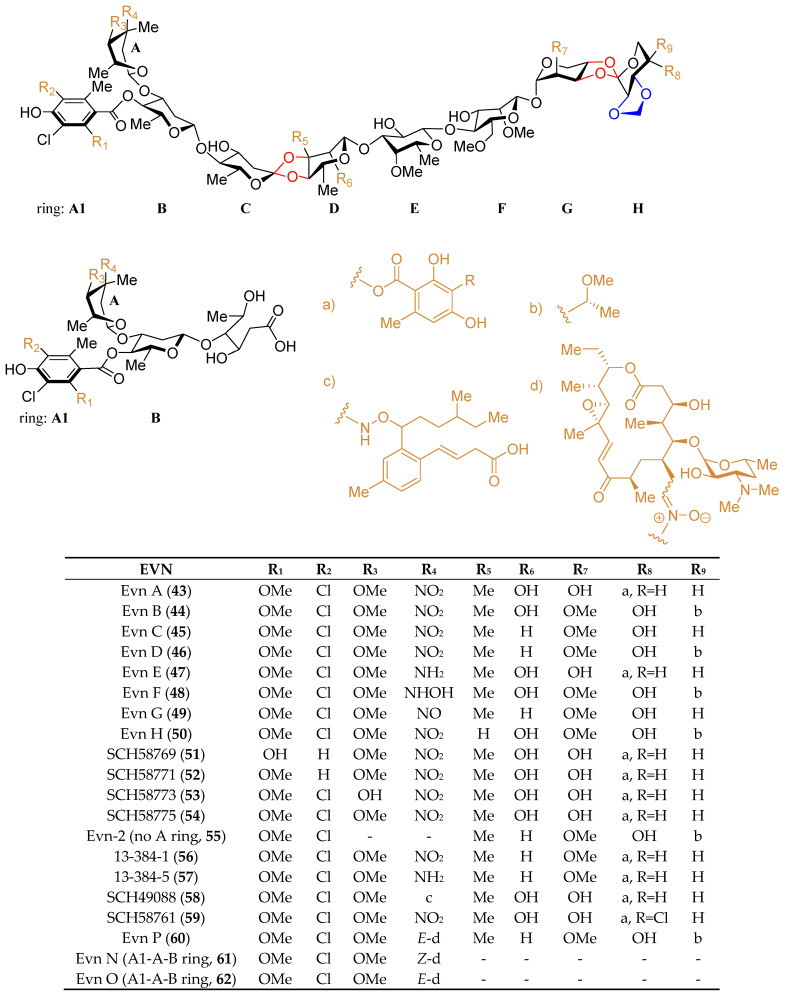
The chemical structures of compounds **43**–**62**. Two orthoester linkages were marked with a red line, while the methylenedioxy bridge was marked with a blue line.

**Figure 9:**
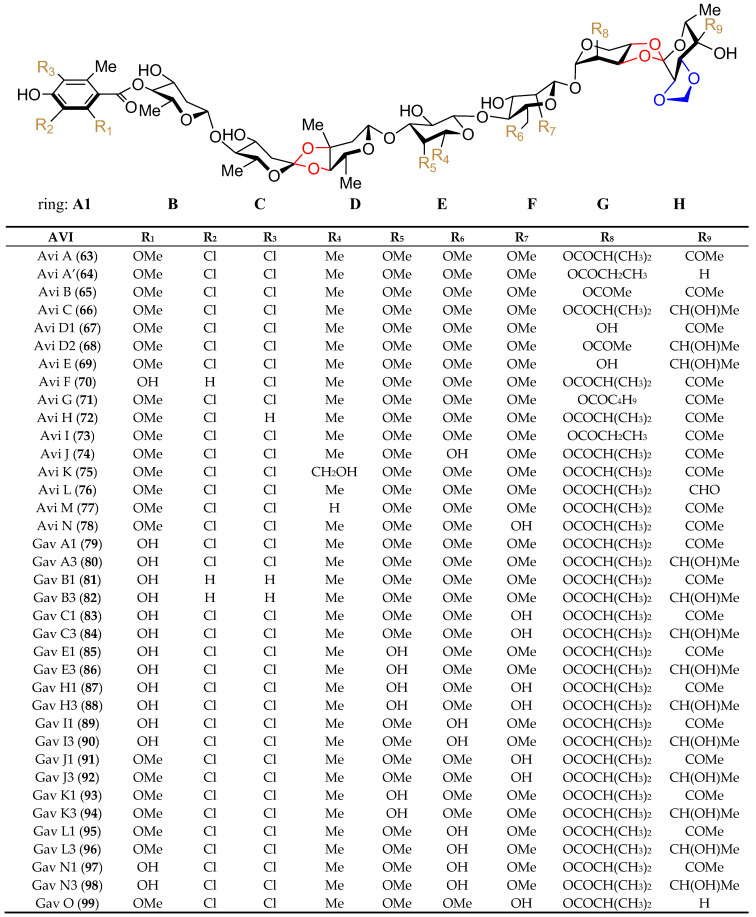
The chemical structures of compounds **63**–**99**. Two orthoester linkages were marked with a red line, while the methylenedioxy bridge was marked with a blue line.

Although hygromycin B (**42**) (Figure 7), produced by *Streptomyces hygroscopicus*, is the first disclosed orthosomycin that possesses one othoster linkage [58], a series of more complicated orthosomycins including EVN and AVI have been published and coined as a new class of antibiotics [59]. Evn D (**46**), the main component of the fermentation broth of *Micromonospora carbonacea* var. *africana*, was identified by Schering-Plough Corporation in 1975 [60]. Since then, Evn D has been utilized as a template for the characterization of other EVN and AVI. Until now, twenty EVN (**43**–**62**) (Figure 8) have been purified from wide- or mutant-type *M. carbonacea* var. *africana* [59,60,61,62,63,71], while thirty-seven AVI (**63**–**99**) (Figure 9) have been isolated from the wide- or mutant-type *Streptomyces viridochromogenes* Tü57 [59,64,72,73,74,75,76,77,78,79,80,81,82]. Structurally, EVN and AVI share the same seven-sugar core (rings B to H) and characteristic substituted partner including two orthoester linkages located between rings C and D and rings G and H and a methylenedioxy bridge attached to ring H. The major differences between EVN and AVI are attributed to the presence (or absence) of the A-ring nitrosugar and the orsellinic acid in EVN (or in AVI).

Amongst the two subgroups of orthosomycins, heptasaccharide AVI (mainly refer to Avi A, **63**) and octasaccharide Evn A (Ziracin, **43**) exhibited the most potent activity against multidrug-resistant bacterial pathogens, including penicillin-resistant streptococci, methicillin resistant staphylococci, and vancomycin resistant enterococci, which was thus developed as lead compounds or clinical drugs for the treatment of antibiotic resistant infections [65,66]. Heptasaccharide AVI premix 10% (MaxusG), firstly developed and produced by Eli Lilly, Bad Homburg, Germany, are widely used as antibiotics in animals [67]. Octasaccharide Evn A (Ziracin) advanced to phase III clinical trials, but the unstated pharmacological concerns prevented its clinical approval [68]. Given that EVN and AVI have a unique mechanism distinguish from other known antibiotics by binding to an exclusive site on bacterial ribosome [69,70], continuous searching for new antimicrobial agents originate from EVN and AVI is a meaningful task. Very recently, reconstruction of the wide-type *M. carbonacea* var. *Africana* targeting the functional related oxidase encoding genes resulted in the discovery of a new intriguing Evn, Evn P (**60**), and its two related hydrolysis products, Evns N (**61**) and O (**62**) [71], which possess either characteristic full-length EVA or partial A1-A-B-ring conjugated to a macrolide rosamicin via a rare nitrone moiety. Particular interestingly, both the full-length and truncated EVA-rosamicin conjugates, Evn P and Evns N, showed significant activities against *S. aureus*, which is almost close to Evn A. Moreover, Evn P displayed a dual targeting of orthogonal binding sites (macrolide and orthosomycin) within the ribosome, which is regarded as a hopeful candidate, generating a new type of hybrid ribosome engaging antibiotic to improve pharmacological properties.

### 4.2. Mode of Action

It is generally understood that the orthosomycin antibiotics EVN and AVI should bind to a unique site on the ribosome, which is different from other ribosome-targeting antibacterial agents currently use in the clinic. In 2016, two research groups disclosed the crystal structures of the EVN and AVI in complex with the bacterial ribosome, which unambiguously demonstrated that both EVN and AVI interacted with arginine residues of ribosomal protein L16 and helices 89 and 91 of the 23S rRNA. This binding site overlaps with the A-site tRNA entrance corridor, which inhibits protein biosynthesis by blocking the binding site of the A-tRNA elbow [69,70].

### 4.3. The Biosynthesis of Avi A

With the consideration of the immense biomedical potential for AVI and EVN, the unique biosynthetic pathways have been investigated, especially for Avi A. In 1997, the Avi A integral biosynthetic gene cluster with a 60 kb (54 ORF) region was discovered from *S. viridochromogenes* Tü57 by constructing a cosmid library, probing with an isotopic-labeled bite gene, and screening with southern hybridization [72,73]. The cluster contains genes proposed to be function as precursor biosynthesis, structural modification, skeletal assembly, resistance, transportation, and regulation in AVI biosynthetic pathway. In details, the precursor synthesis genes include *aviN*, *aviM*, *aviD*, *aviE1*-*aviE3*, *aviS*, *aviB1*, and *aviB2*. Structural modification genes contain five types, that is, methyltransferase genes *aviG1*-*aviG6*, epimerase genes *aviQ1*-*aviQ3*, ketoreductase genes *aviT* and *aviZ1*-*aviZ3*, halogenase gene *aviH*, and *S*-adenosylmethioninase gene *aviX12*. The skeletal assembly genes include glycosyltransferase genes *aviGT1*-*aviGT4* and hydroxylase genes *aviO1*-*aviO3.* The former is putatively responsible for the assembly of heptasaccharide chain, while the latter may participate in the formation of *O*-glycosidic and orthoester bonds. In addition, *aviRa* and *aviRb* perhaps be related to AVI resistance, *aviABC1* and *aviABC2* are involved in AVI transport, while *aviC1* and *aviC2* may act as positive regulators (Table 2).

**Figure 10:**
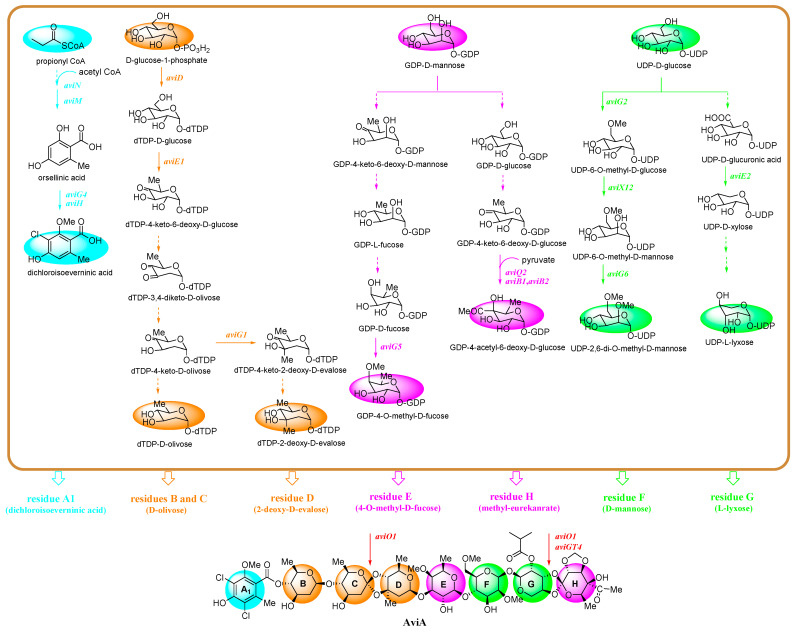
The proposed Avi A biosynthetic pathway (the blue branch is for dichloroisoeverninic acid (moiety A), the orange one is for *d*-olivose (residues B and C) and 2-deoxy-*d*-evalose (residue D), the pink branch is for *d*-fucose (residue E) and methyleurekanate (residue H), while the green one is for *d*-mannose (residue F) and *l*-lyxose (residue G); the content in the square bracket represents the published year of genes. The genes in the Avi A biosynthetic gene cluster mentioned above are experimentally defined by proteomic studies.

As mentioned above, the backbone of Avi A is composed of a terminal dichloroisoeverninic acid unit (residue A) and its attachment to a heptasaccharide chain consisting of *d*-olivose (residues B and C), 2-deoxy-*d*-evalose (residue D), *d*-fucose (residue E), *d*-mannose (residue F), *l*-lyxose (residue G), and methyleurekanate (residue H). Due to the multiple-ring structure of Avi A, its biosynthetic pathway is relatively complicated. Up to now, aided with the bioinformatics analysis, some of the key enzymes in the Avi A biosynthesis pathway have been successfully elucidated by analysis of the corresponding Avi derivatives, gavibamycins (Gav, **79**–**99**), in situ gene knockout and complementation strategy, heterologous expression systems, such as *S. lividans* TK24, *S. coelicolor* CH999, *Streptomyces* TK66, etc., or feeding experiments [72,73,74,75,76,77,78,79,80,81,82]. Herein, four main initial precursors, including propionyl-CoA, *d*-glucose-1-phosphate, GDP-*d*-mannose, and UDP-*d*-glucose, are summarized in Figure 10. Accordingly, the biosynthesis of the skeletal assembly of the eight moieties A-H in Avi A is divided into four main branches distinguished from each other with colors as shown in Figure 10. Of all the branches, the biosynthesis for moieties A (blue) and F (sub-branch, green) have been fully or almost fully elucidated. For the biosynthesis of moiety A, starting from propionyl-CoA, iterative type I polyketide synthase AviM [72], is responsible for the synthesis of orsellinic acid, which is further catalyzed by methyltransferase AviG4 [73] and halogenase AviH [73] to produce the complete dichloroisoeverninic acid moiety. For the biosynthesis of moieties B–D (orange), the starting precursor, glucose-1-phosphate, is converted to dTDP-*d*-olivose and dTDP-2-deoxy-*d*-evalose, respectively, and three enzymes including dTDP-glucose synthase AviD [72], dTDP-glucose 4,6-dehydratase AviE1 [72], and *C*-methyltransferase AviG1 [74] have been discovered to anticipate the biosynthesis of rings B-D. For the biosynthesis of residues E and H (pink), GDP-*d*-mannose is their common precursor. Hydroxylase AviO2 [75] combined with pyruvate dehydrogenase AviB1 and AviB2 [75], a heterotetrameric complex protein (*α*2*β*2 chains), catalyze the conversion of pyruvate to an acetyl carbanion at C4 in eurekanate moiety (ring H), while *O*-methyltransferase AviG5 [76] is the answer for the methylation of the C4 hydroxyl group in GDP-*d*-fucose in ring E; for the biosynthesis of residues F and G (green), their common precursor is UDP-*d*-glucose. Decarboxylase AviE2 [77] converts UDP-*d*-glucuronic acid to UDP-*d*-xylose in ring G, while AviX12 [78], a [Fe-S] cluster containing radical AdoMet enzyme catalyzing an unusual epimerization reaction, together with two O-methyltransferase AviG2 and AviG6 [76], complement the whole biosynthesis of ring F.

As we know, the residues A-H in Avi A are associated by orthoester linkages, glycosidic linkages, ester linkages, and a methylenedioxy bridge. Until now, only the partial linkage between rings G and H have been proved to be catalyzed by glycosyltransferase AviGT4 [77], while the others still remain unknown. Orthoester group as a characteristic pharmacophore of octasaccharides is crucial to the biomedical application of Avi A. Therefore, clarifying the formation of the orthoester group is a continuous target for scientists. Recently, McCulloch [79] have identified a conserved group of nonheme iron, *α*-ketoglutarate-dependent oxygenases, including AviO1 of AVI and its two homologues, EvdO1 and EvdO2 of EVN and HygX of hygromycin B. Analysis of the high-resolution crystal structures reveals that an orientation of one glycosidic linkage of oxygenase HygX is consistent with metal-catalyzed hydrogen atom abstraction from substrate, supporting that AviO1 participates the catalysis of the orthoester linkages. In addition, two resistances, rRNA-methyltransferase AviRa and AviRb, two regulators, ABC transporters AviABC1 and AviABC2 [80], and two transcriptional activators, AviC1 and AviC2 [81] are also reported. Regarding the Avi A contributes to poor water solubility and difficulties in drug development perhaps ascribed to its multiple methyl groups [76,82], fully understanding the Avi A biosynthetic pathway will provide useful information and contribute to produce novel derivatives with improved polarity and pharmacokinetic properties.

## 5. Summary and Perspectives

Despite the fact that the first oligosaccharide (hygromycin B) was described in 1958, this family of compounds has received sufficient attention only recently. A small number of bioactive oligosaccharides have been examined in clinical trials or introduced into the drug market for the treatment of infectious diseases and metabolic disorders, such as acarbose (α-amylase inhibitor), hygromycin B and Avi A (veterinary drugs), and Evn A (antimicrobial agent). The most widely used oligosaccharide is acarbose, a pseudotetrasaccharide exhibiting an inhibitory effect on intestinal α-glucosidases such as α-amylase, sucrase, maltase, and isomaltase. It is worth noting that the higher molecular weight acarviostatins, exhibiting more potent activities against α-glucosidases than acarbose, were isolated from the culture broths of various members of the genus *Streptomyces*, which suggested that this class of compounds could be widely distributed in the marine and terrestrial bacteria. More importantly, the culturable marine-derived actinomycetes are becoming an outstanding source for the discovery of new aminooligosaccharides.

Owing to the structural complexity of oligosaccharide secondary metabolites, especially the fact that each of them has multiple monosaccharides, it is very challenging to obtain those compounds and their analogues by chemical synthesis, which turned the research interest of medical chemists toward their biosynthesis. The efforts on the biosynthesis of oligosaccharide secondary metabolites have revealed several closely related novel biosynthetic pathways exclusively produced by microorganisms. The decipherment of the metabolic pathways of oligomeric natural products is beneficial for shedding more light on their mode of formation and distinguishing biosynthetic features. In the meantime, the wealth of knowledge acquired from research on oligosaccharides biosynthesis has provided new opportunities to produce novel derivatives by means of combinatorial biosynthesis or synthetic biology approaches, which can expand the diversity of oligosaccharides to some extent. In addition, the advances in DNA sequencing, bioinformatics and omics, and molecular genetics in recent years, have enabled the expansion of our understanding of oligosaccharides biosynthesis and the titer improvement of certain microbial pharmaceutical products, which contributes to produce many more of oligomeric analogues for structure–activity relationship studies.

## Figures and Tables

**Table 1:** The deduced functions of the acarviostatins biosynthetic genes in *S. coelicoflavus* ZG0656.

Genes	Amino Acids	Identity/Similarity to the *acb*-Cluster	Proposed Function
SctZ2	1619	50%/63% homologous to AcbZ	*α*-amylase
SctZ1	1042	47%/59% homologous to AcbZ	*α*-amylase
SctE2	565	25%/39% homologous to AcbD	*α*-amylase
SctC2	352	no homologue	LacI family transcriptional regulator
SctE1	553	25%/36% homologous to AcbE	*α*-glucosidase
SctG	301	26%/51% homologous to AcbG	ABC transporter membrane protein
SctF	335	29%/50% homologous to AcbF	ABC transporter membrane protein
SctH	424	25%/37% homologous to AcbH	ABC transporter membrane protein
SctC1	342	no homologue	malR-like regulator
SctB	325	59%/70% homologous to AcbB	dTDP-glucose 4,6-dehydratase
SctA	357	43%/59% homologous to AcbA	dTDP-glucose synthase
SctV	417	75%/83% homologous to AcbV	dTDP-4-keto-6-deoxy-glucose 4-aminotransferase
SctW	341	62%/77% homologous to AcbW	ABC transporter permease protein
SctX	274	49%/68% homologous to AcbX	ABC transporter permease protein
SctY	268	57%/75% homologous to AcbY	ABC transporter permease protein
SctU	487	42%/51% homologous to AcbU	1-*epi*-valienol-7-phosphate-1-kinase
SctS	691	62%/71% homologous to AcbS	glycosyltransferase
SctR	354	70%/80% homologous to AcbR	1-*epi*-valienol-1,7-bisphosphate-1-adenylyltransferase
SctK	308	48%/60% homologous to AcbK	acarbose-7-kinase
SctI	1027	47%/59% homologous to AcbI	putative glycosyltransferase
SctQ	702	55%/66% homologous to AcbQ	putative acarbose 4-alpha-glucanotransferase
SctC	393	39%/52% homologous to AcbC	2-*epi*-5-*epi*-valiolone synthase
SctJ	138	no homologue	2-epimerase
SctM	350	31%/43% homologous to AcbM	C_7_-cyclitol-7-kinase
SctO	324	no homologue	putative 2-*epi*-5-*epi*-valiolone dehydratase
SctT	464	no homologue	unknown
Scim	112	no homologue	*α*-amylase

**Table 2:** The proposed functions of the AVI biosynthetic genes in *S. viridochromogenes* Tü57.

Genes	Encoded Enzymes	Proposed Function
*avi N*	ketoacyl synthase III homologues	controlling the starter unit for orstainic acid biosynthesis
*avi M*	type I polyketide synthase	orstainic acid biosynthesis
*avi D*	dTDP-glucose synthase	starter enzyme for residues B, C, D biosynthesis
*avi E1*, *aviS*	dTDP-glucose-4,6-dehydratase	residues B, C, D biosynthesis
*avi E2*	UDP-glucuronic acid decarboxylase	starter enzyme for residue G biosynthesis
*avi E3*	GDP-mannose-4,6-dehydratase	starter enzyme for residue E biosynthesis
*avi B1*, *avi B2*	pyruvate dehydrogenase (α_2_β_2_ chains)	residue H biosynthesis
*avi G1*	*C*-methyltransferase	residue D biosynthesis
*avi G5*	*O*-methyltransferase	C-4 methylation of residue E
*avi G2*, *avi G6*	*O*-methyltransferase	C-2, C-6 methylation of residue F
*avi G3*	*O*-methyltransferase	residue H methylation
*avi G4*	*O*-methyltransferase	residue N methylation
*avi Q1*-*avi Q3*	UDP-glucose-4-epimerase	epimerization of oligosaccharides
*avi Z1*	ketoreductase	residues D and E synthesis
*avi T*	ketoreductase	residues B-D synthesis
*avi Z3*	ketoreductase	residues B and C synthesis
*avi Z2*	ketoreductase	residues D and E synthesis
*avi H*	halogenase	halogenated residue A
*avi X12*	S-adenosylmethioninase	activated residues F and G
*avi O1*, *avi O3*	hydroxylase	formation of orthoester bond and glycosidic bond
*Avi O2*	hydroxylase	residue H biosynthesis
*avi GT1*-*avi GT3*	glycosyltransferase	assembly of heptasaccharide chain
*avi GT4*	glycosyltransferase	conjugation between residues G and H
*avi C1*, *avi C2*	regulator	AVI positive regulator
*avi Ra*, *avi Rb*	rRNA methyltransferase	AVI resistance
*avi ABC1*, *avi ABC2*	ABC transporter, ATP binding protein	AVI antibiotic transport

## Data Availability

Not applicable.
